# High Resolution Ultrasound for the Assessment of Melasma: Experience With 1064 nm Q-Switched Nd:YAG Laser

**DOI:** 10.7759/cureus.98719

**Published:** 2025-12-08

**Authors:** Laura Trujillo Ramírez, María Fernanda Meza Corso, Claudia Gonzalez, Julio Roberto Amador, Angie Holguín, César González

**Affiliations:** 1 Dermatology, Private Practice, Bogotá, COL; 2 Dermatology, Universidad Libre, Cali, COL; 3 Radiology/Dermatology Ultrasound, Rosario University, Bogotá, COL; 4 Dermatology, Clinica el Country, Bogotá, COL; 5 Dermatology, Universidad el Bosque, Bogotá, COL

**Keywords:** doppler, high resolution ultrasound, melasma, melasma treatment, nd:yag laser, ultrasound doppler

## Abstract

Melasma is a challenging chronic pigmentary disorder that holds considerable significance for both patients and dermatologists. Monitoring therapeutic outcomes is essential for optimizing treatment strategies. This study aimed to apply high-resolution dermatologic ultrasound (HRDU) to evaluate skin changes in melasma patients and document the effects observed following the application of collimated and fractional forms of 1064 nm Q-switched Nd:YAG (QSNY) laser therapy.

In a prospective case series conducted from December 2024 to February 2025 at a private dermatology clinic, three patients with melasma who were indicated for QSNY laser treatment were enrolled. Each patient underwent three high-resolution dermatologic ultrasound examinations: the first before initiating QSNY laser therapy, the second 24-48 hours after the initial session, and the third three weeks following the third session. Demographic data of the sample, Melasma Severity Index (MSI) score, ultrasound grayscale, and color Doppler features were recorded and analyzed.

At baseline, all patients demonstrated a subepidermal low-echogenic band (SLEB), follicular dilation, and increased vascularization in the forehead and/or cheek regions. Following the laser sessions, we observed a decrease in the SLEB, an improvement in echogenicity, and an increase in dermal capillarity. These findings provide innovative documentation of skin changes in melasma, including subclinical inflammation and solar elastosis, and highlight the potential of low fluence, fractionated QSNY laser to reduce them. HRDU emerges as a valuable tool to confirm and monitor these therapeutic benefits, supporting its role in personalized treatment strategies for melasma.

## Introduction

Melasma is an acquired, chronic, and benign disorder, consisting of irregular pigmentation of the skin [1]. In Latin America, it is a frequent reason for dermatologic consultation, affecting up to 10% of the population [2]. Clinically, it presents as brown or gray hyperpigmented macules of varying intensity, typically occurring on photoexposed areas, with a strong predilection for the face, particularly the forehead, cheeks, upper lip, and nasal dorsum [1]. As a result, melasma can substantially impact individuals’ self-esteem and overall quality of life [3].

The pathophysiology of melasma is complex, and one of the predominant mechanisms related to its appearance and relapses is chronic sun exposure [4]. Other related factors include estrogen exposure, thyroid hormone alterations, and a family history of melasma [5]. Histologically, melasma exhibits skin alterations similar to those seen in photoaging, including solar elastosis, basement membrane disruption, and increased vascularization [6]. Treatment strategies encompass conservative measures, such as photoprotective agents and camouflage, as well as active treatment, including topical depigmenting agents, tranexamic acid, and energy-based technologies designed for pigment destruction, such as laser therapy [7-9].

Q-switched Nd:YAG (QSNY) laser delivers high-intensity energy through ultra-short pulses. This generates a photoacoustic effect that disrupts melanosome structures and reduces melanocyte dendrites, primarily in the deep epidermis and dermal layers. This mechanism decreases pigmentation with minimal adverse effects, such as residual hyperpigmentation or punctual hypopigmentation [10]. Additionally, the fractional modality of this laser induces the formation of epidermal and dermal vacuoles, corresponding to the effects of laser-induced optical breakdown (LIOB) and/or laser-induced cavitation (LIC) [11]. These effects lead to increased collagen density and dermal elastic fibers, resulting clinically in reduced pigmentation and improved skin texture [11,12].

High-resolution dermatologic ultrasound (HRDU) is a non-invasive tool that enables real-time epidermis, dermis, and subcutaneous tissue evaluation using high-resolution transducers with frequencies ranging from 15 to 70 MHz [13,14]. Its application in dermatology has been widely described in non-tumorous inflammatory pathologies [15,16]. More recently, it has been employed to evaluate the therapeutic effects of anti-aging treatments [17]. In both scenarios, significant differences have been observed in parameters such as the thickness and echogenicity of individual skin layers, subepidermal band thickness, adnexal structure size, and microvascular features [16,17]. However, existing literature has not described these ultrasound findings in the context of melasma.

Consequently, it could be interesting to document the presence of subclinical inflammatory activity that could predict therapeutic response or identify patients at risk for a poorer prognosis. Currently, both topical therapies and energy-based technologies are assessed primarily through clinical evaluation. This study aims to investigate skin changes in melasma using HRDU after treatment with collimated and fractional 1064 nm QSNY laser therapy.

## Case presentation

A prospective case series study was performed at a private dermatology clinic between December 2024 and February 2025. This study was approved by the Institutional Scientific Committee of Clinica del Country in Bogotá, Colombia. Three patients with clinically and dermoscopically confirmed melasma were included. Exclusion criteria included any topical or laser therapy within the previous three months, as well as the presence of any dermatoses in the treated area that could interfere with clinical or HRDU evaluation. All patients signed an informed consent for participation in the study and publication of their images. 

Patients were treated by a single laser expert (LT). Each patient underwent three laser sessions, separated by at least 20 days, combining low-fluence and fractional QSNY laser (Hollywood Spectra XT, Lutronic corporation, Goyang, Korea), using the MDF handpiece with fluence of 1.8 to 2 J/cm^2^ with 6-8 mm spot size, 10 Hz frequency in two passes, and collimated handpiece: fluence 0.9 to 2.6 J/cm^2^ with 6-8 mm spot size, 10 Hz frequency focused on the macule. For each patient, HRDU was performed before, up to 48 hours after the first laser session, and three weeks after the third QSNY laser session. The ultrasound device used was a Venue System (General Electric Health Systems, Waukesha, WI) with a linear probe up to 18 MHz. All ultrasound exams were performed by the same trained radiologist (CG) with over than 13 years of experience in dermatologic ultrasound.

The ultrasound protocol included the study of five facial subregions: forehead, right malar, left malar, chin, and nose dorsum. All ultrasound studies followed the international guidelines for performing dermatologic ultrasound examinations [13]. Cutaneous layers morphology, pilosebaceous unit characteristics were recorded and analyzed. This included epidermal alterations, dermal echogenicity, vascularity and thickness, hair follicle dilatation, and sebaceous gland hypertrophy. Cutaneous vascularity was studied with color Doppler. It was considered hypervascularity when three or more vessels were detected in the dermis. 

Demographic data of the sample, Melasma Severity Index (MSI) score [18], ultrasound grayscale, and color Doppler features were analyzed using descriptive statistical methods. 

Results

Three female patients aged 34-53 years who met the inclusion criteria were included in the study. All had completed university-level education and had no significant comorbidities. All patients exhibited involvement of both malar regions, nasal dorsum, and forehead. One patient had Fitzpatrick phototype III, and two had phototype IV. According to the palmar crease pigmentation scale, all were categorized as having a low risk of post-inflammatory hyperpigmentation [19]. 

In accordance with the clinical protocol, all participants underwent HRDU before laser therapy and again 48 hours after the first session. All completed the three planned laser sessions and the post-treatment clinical evaluation. Two of the three patients underwent the final HRDU assessment three weeks after the last laser session. One patient (patient 3) was unable to complete the final HRDU due to personal circumstances unrelated to the intervention (Table 1).

**Table 1:** High-resolution dermatologic ultrasound assesment The second ultrasound assessment was conducted 48 hours following the first laser session. The final ultrasound was performed three weeks after the third session. Dermal thickness was measured in millimeters. Other parameters were considered present or absent according to the ultrasound evaluation performed (✓: present, X: absent) SLEB: subepidermal low-echogenic band

High-resolution dermatologic ultrasound assessment																
		Initial					Second					Final				
Patient	Parameter	Forehead	Right malar	Left malar	Chin	Nose dorsum	Forehead	Right malar	Left malar	Chin	Nose dorsum	Forehead	Right malar	Left malar	Chin	Nose dorsum
1	Epidermal Undulation	✓	✓	✓	X	✓	✓	X	✓	X	X	X	X	X	X	X
	SLEB	✓	✓	✓	X	✓	✓	✓	✓	X	X	X	X	X	X	X
	Dermal hypoechogenicity	✓	X	X	✓	✓	✓	X	X	✓	X	X	X	X	X	X
	Dermal thickness (mm)	0.7	0.9	0.9	1.2	0.4	0.7	1.3	0.9	1.6	1	0.7	1.2	1.8	2	1.3
	Doppler assessment	Normal	Normal	Normal	Increased	Increased	Increased	Normal	Normal	Increased	Normal	Normal	Normal	Normal	Normal	Normal
	Follicular dilation	✓	✓	✓	X	X	X	✓	X	X	X	X	X	X	X	X
	Sebaceous gland hypertrophy	X	✓	X	X	X	X	✓	X	X	X	X	X	X	X	X
2	Epidermal undulation	✓	✓	X	X	X	✓	✓	✓	✓	X	✓	X	X	X	X
	SLEB	✓	✓	✓	X	X	X	X	X	X	X	X	X	X	X	X
	Dermal hypoechogenicity	✓	✓	X	X	X	✓	X	X	X	X	X	X	X	X	X
	Dermal thickness (mm)	0.9	0.6	0.6	0.6	0.5	0.5	1	1.2	1	0.4	0.8	1.4	1.2	2	0.5
	Doppler assessment	Normal	Normal	Normal	Normal	Normal	Normal	Normal	Increased	Increased	Normal	Normal	Normal	Normal	Increased	Normal
	Follicular dilation	✓	✓	X	X	X	X	✓	✓	X	X	X	✓	✓	X	X
	Sebaceous gland hypertrophy	X	X	X	X	X	X	X	X	X	X	X	X	X	X	X
3	Epidermal undulation	X	X	✓	X	X	X	X	✓	X	X					
	SLEB	X	✓	✓	X	X	X	✓	✓	X	X					
	Dermal hypoechogenicity	X	X	X	X	X	X	X	X	X	X					
	Dermal thickness (mm)	2.4	1.8	1.6	1.4	1.2	2.4	1.8	1.6	1.8	1.2					
	Doppler assessment	Normal	Normal	Normal	Normal	Normal	Increased	Normal	Normal	Increased	Normal					
	Follicular dilation	X	✓	X	X	X	X	✓	X	X	X					
	Sebaceous gland hypertrophy	X	X	X	X	X	X	X	X	X	X					

**Figure 1:**
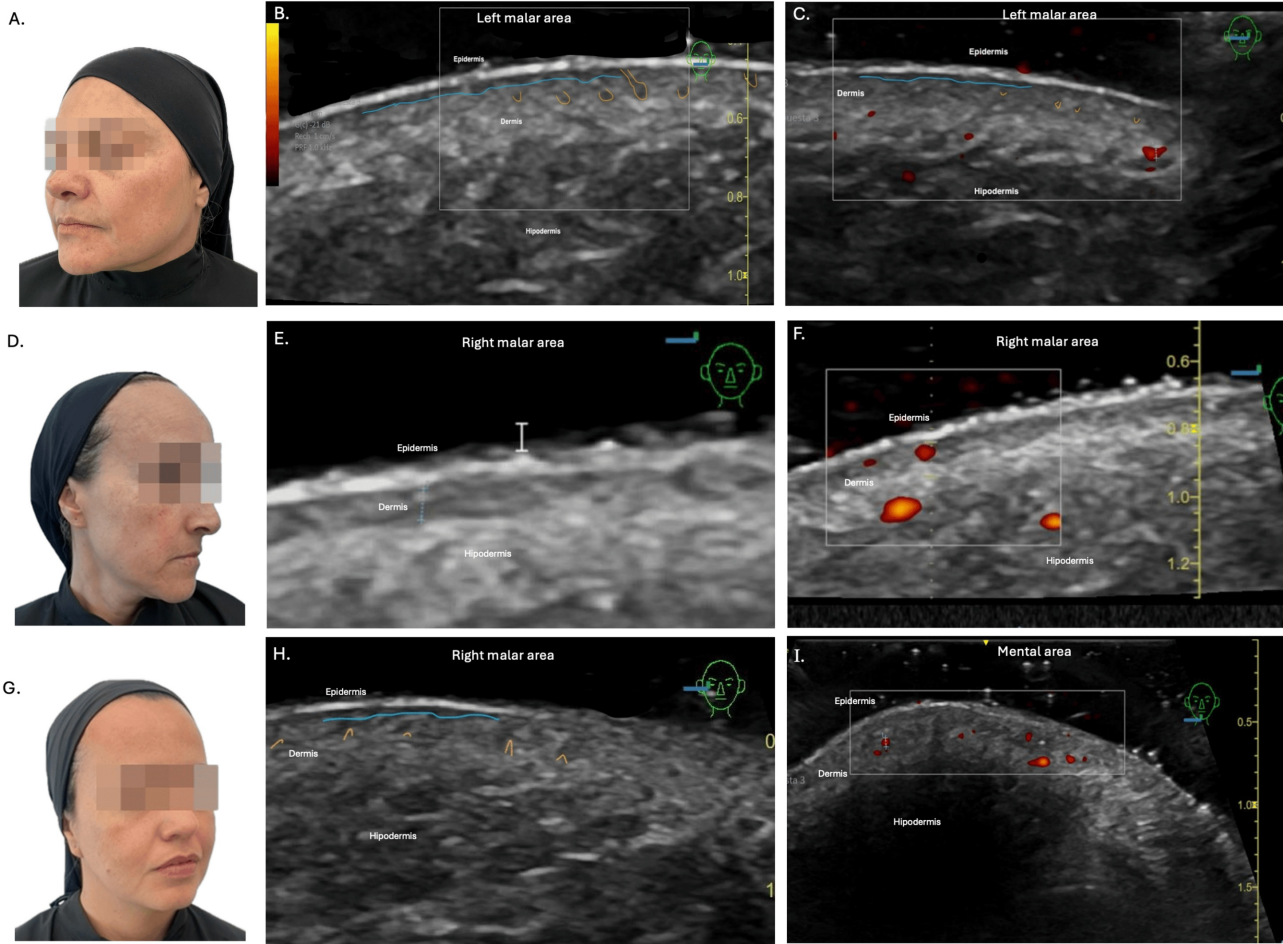
Initial clinical and high-resolution dermatologic ultrasound assesment A. Patient 1. Initial clinical evaluation. Mixed melasma predominantly in the forehead and cheek areas B. And C. Patient 1. Initial Ultrasound. Grayscale images: blue line: SLEB; yellow line: follicular dilatation. Power Doppler: red areas represent dermal micro-vascularization D. Patient 2. Initial clinical evaluation. Mixed melasma predominantly in the forehead and cheek areas E. And F. Patient 2. Initial Ultrasound. Grayscale images and Power Doppler: red areas represent dermal micro-vascularization G. Patient 3. Initial clinical evaluation. Mixed melasma predominantly in the forehead and cheek areas H. And I. Patient 3. Initial Ultrasound. Grayscale images: blue line: SLEB; yellow line: follicular dilatation. Power Doppler: red areas represent dermal micro-vascularization SLEB: subepidermal low-echogenic band

Initial ultrasound evaluation revealed a subepidermal low-echogenic band (SLEB) in melasma-affected regions in all patients. Patients 1 and 3 exhibited this finding in the malar and forehead regions, whereas patient 2 also showed it in the nasal dorsum. Additional shared findings included epidermal undulation, dermal hypoechogenicity, and follicular dilation. Sebaceous gland hypertrophy was noted only in patient 1. The average total dermal thickness was 0.82 mm in patient 1, 0.64 mm in patient 2, and 1.68 mm in patient 3. Mean vascular diameter was 0.30 mm, 0.26 mm, and 0.44 mm, respectively. Doppler assessment revealed increased vascular flow in the chin and nasal dorsum of patient 1, while this parameter remained within normal limits in the other patients (Figure 1).

**Figure 2:**
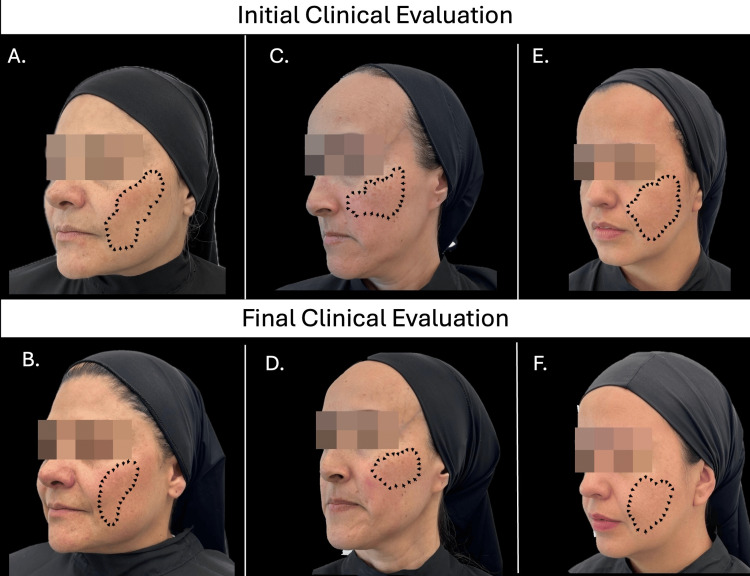
Clinical improvement in patients A. Patient 1. Initial clinical evaluation. Mixed melasma predominantly in the forehead and cheek areas. MSI: 16 B. Patient 1. Final clinical evaluation. Mixed melasma predominantly in the forehead and cheek areas. MSI: 3.4 C. Patient 2. Initial clinical evaluation. Mixed melasma predominantly in the forehead and cheek areas. MSI: 6.6 D. Patient 2. Final clinical evaluation. Mixed melasma predominantly in the forehead and cheek areas. MSI: 1.8 E. Patient 3. Initial clinical evaluation. Mixed melasma predominantly in the forehead and cheek areas. MSI: 14.6 F. Patient 3. Final clinical evaluation. Mixed melasma predominantly in the forehead and cheek areas. MSI: 6.6 MSI: Melasma Severity Index

A second HRDU performed 48 hours after the first laser session revealed acute changes, including decreased SLEB visibility in patients 1 and 2, and resolution of hypoechogenicity in the nasal dorsum (patient 1) and malar region (patient 2). Doppler evaluation showed variable responses: patient 1 exhibited normalization of vascular flow in the nasal dorsum, while patient 2 and patient 3 demonstrated increased Doppler signal in the forehead, malar, and chin regions compared to baseline. Three weeks after completing the three-session laser protocol, clinical evaluation showed an average reduction in MSI of 68.76% (SD: ± 12.78) (Figure 2).

**Figure 3:**
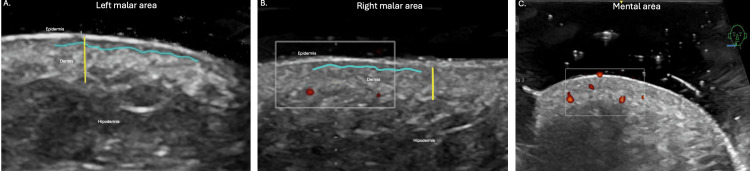
Final high-resolution dermatologic ultrasound assesment A. Patient 1. Ultrasound 3 weeks after the third laser session. Grayscale images: blue line: decrease in SLEB; yellow line: increase in dermal thickness B. Patient 2. Ultrasound 3 weeks after the third laser session. Grayscale images: blue line: decrease in SLEB; yellow line: increase in dermal thickness. C. Patient 3. Ultrasound 48 hours after the laser session. Grayscale images: increase in dermal capillary diameter due to reparative process, improvement of dermal echogenicity SLEB: subepidermal low-echogenic band

Doppler assessment showed variable outcomes: in patient 1, vascular flow normalized in the chin and nasal dorsum regions, while in patient 2, the Doppler signal increased in the chin region, despite being initially within normal limits. Final ultrasound assessment revealed an increase in dermal thickness; other findings, such as SLEB, epidermal undulation, dermal hypoechogenicity, and follicular dilation, were no longer detectable in the patients who completed the follow-up (Figure 3).

## Discussion

HRDU is a non-invasive technique that enables real-time evaluation of skin structures and has multiple applications in clinical and aesthetic dermatology [20]. While melasma is typically diagnosed through clinical evaluation, HRDU has not been described as a diagnostic tool for this condition. Nevertheless, the ultrasonographic changes observed in this study suggest that some features correlate with histological and reflectance confocal microscopy findings previously reported in the literature, including solar elastosis and vascular pattern alterations [6,21]. Solar elastosis is a histological feature characterized by the degeneration and aggregation of elastotic fibers in the dermis [22]. This alteration is more pronounced in melasma lesions compared to unaffected areas, supporting the role of chronic ultraviolet radiation (UVR) exposure in the pathogenesis of the condition [23,24]. In HRDU, solar elastosis is strongly associated with the presence of SLEB [20]. In this study, this feature was identified in at least one anatomical area in all patients during the baseline ultrasound.

Increased dermal vascularity has been linked to inflammatory activity in hyperpigmented areas [25]. This finding has greater specificity for melasma and may help differentiate it from other pigmentary or photoaging-related conditions [26,27]. Moreover, in reflectance confocal microscopy studies, dermal vascular dilation has been proposed as a defining criterion for the mixed subtype of melasma [21]. In this study, all patients were clinically and Wood’s lamp-diagnosed with mixed-type melasma, and initial HRDU assessments revealed increased dermal vascularity in every case. The use of Doppler imaging in HRDU allows vascular flow assessment without interference from melanin, a major limitation of dermoscopy and digital imaging analysis systems [25]. While confocal microscopy and optical coherence tomography may also overcome this issue [21,28], their limited accessibility due to cost and availability restricts widespread use in clinical settings.

In this study, HRDU was also applied to determine whether subclinical changes could be identified following low-fluence QSNY laser treatment. A reduction in SLEB and an increase in dermal thickness were observed, potentially reflecting collagen fiber reorganization induced by the laser. This dermal remodeling effect has also been visualized by reflectance confocal microscopy, where fine hyper-refractile dermal matrix changes were reported [29]. Longer-term dermal effects have been documented in histologic studies where pigment clearance has served as the primary endpoint, with a reduction in dermal melanophages observed one month after multiple laser sessions [30]. In this study, HRDU, performed three weeks after treatment, confirmed persistence of SLEB reduction, absence of epidermal undulation, dermal hypoechogenicity, and follicular dilation, although additional studies are necessary to establish whether this may serve as a biomarker of therapeutic response.

This study has certain limitations, including the small sample size, single-center design, and short follow-up registration. However, after a detailed review of the literature, we present the first application of HRDU in assessing melasma within a Latin American population, evaluating the effects of combining low fluence, fractionated QSNY laser. Our findings support the use of non-invasive, accessible tools to enhance the evaluation of therapeutic strategies in melasma, a condition that remains challenging to manage.

## Conclusions

As a non-invasive modality, HRDU holds potential for clinical application in sensitive aesthetic areas such as the face and may become a valuable tool in personalized treatment planning for melasma. The detection of SLEB in melasma-affected areas, along with its correlation to solar elastosis, reinforces the role of chronic UVR exposure in the disease pathogenesis. Moreover, HRDU provides valuable insights into dermal and vascular features that may otherwise go unnoticed with conventional clinical tools. This comprehensive characterization may support more personalized treatment strategies by helping to identify patients who could benefit from therapies targeting basement membrane repair and others who may respond better to vascular-targeted interventions. Although this study has some limitations, our findings enhance the evaluation and monitoring of therapeutic strategies in melasma, a challenging and recurrent condition. Future studies with larger cohorts and longer follow-ups are warranted to confirm these preliminary findings and to further establish the utility of HRDU as a diagnostic, predictive tool for treatment response in melasma.
